# Genome-wide DNA methylation analysis of *Haloferax volcanii* H26 and identification of DNA methyltransferase related PD-(D/E)XK nuclease family protein HVO_A0006

**DOI:** 10.3389/fmicb.2015.00251

**Published:** 2015-04-08

**Authors:** Matthew Ouellette, Laura Jackson, Scott Chimileski, R. Thane Papke

**Affiliations:** Department of Molecular and Cell Biology, University of ConnecticutStorrs, CT, USA

**Keywords:** DNA methylation, restriction modification system, haloarchaea, *Haloferax volcanii*, *Halobacteria*, methylome, PD-(D/E)XK nuclease, methylation

## Abstract

Restriction-modification (RM) systems have evolved to protect the cell from invading DNAs and are composed of two enzymes: a DNA methyltransferase and a restriction endonuclease. Although RM systems are present in both archaeal and bacterial genomes, DNA methylation in archaea has not been well defined. In order to characterize the function of RM systems in archaeal species, we have made use of the model haloarchaeon *Haloferax volcanii*. A genomic DNA methylation analysis of *H. volcanii* strain H26 was performed using PacBio single molecule real-time (SMRT) sequencing. This analysis was also performed on a strain of *H. volcanii* in which an annotated DNA methyltransferase gene *HVO_A0006* was deleted from the genome. Sequence analysis of H26 revealed two motifs which are modified in the genome: C^m4^TAG and GCA^m6^BN_6_VTGC. Analysis of the Δ*HVO_A0006* strain indicated that it exhibited reduced adenine methylation compared to the parental strain and altered the detected adenine motif. However, protein domain architecture analysis and amino acid alignments revealed that HVO_A0006 is homologous only to the N-terminal endonuclease region of Type IIG RM proteins and contains a PD-(D/E)XK nuclease motif, suggesting that HVO_A0006 is a PD-(D/E)XK nuclease family protein. Further bioinformatic analysis of the *HVO_A0006* gene demonstrated that the gene is rare among the *Halobacteria*. It is surrounded by two transposition genes suggesting that *HVO_A0006* is a fragment of a Type IIG RM gene, which has likely been acquired through gene transfer, and affects restriction-modification activity by interacting with another RM system component(s). Here, we present the first genome-wide characterization of DNA methylation in an archaeal species and examine the function of a DNA methyltransferase related gene *HVO_A0006*.

## Introduction

Restriction-modification (RM) systems in bacteria and archaea provide individuals the ability to recognize self from non-self DNA and function as host defense mechanisms, protecting their genomes from foreign DNA invasion (Arber and Dussoix, 1962; Meselson et al., 1972; Vasu and Nagaraja, 2013). These systems are composed of a pair of enzymes: a restriction endonuclease and its cognate methyltransferase, which recognize identical short DNA sequences known as the recognition sequence. Endonucleases typically cleave dsDNA by hydrolyzing the phosphodiester bonds at one location after detection of an unmethylated recognition sequence (Loenen et al., 2014b). Methyltransferases catalyze a reaction that transfers a methyl group from a cofactor, S-adenosyl-L-methoinine (AdoMet), and modifies a specific nucleotide base (Wilson and Murray, 1991; Blumenthal and Cheng, 2002). Three separate types of methylated bases have been identified: N6 methyl-adenine (m^6^A), C5 methyl-cytosine (m^5^C), and M4 methyl-cytosine (m^4^C) (Wion and Casadesus, 2006). In nearly all cases, the addition of a methyl group to a base in the recognition sequence by the cognate methyltransferase prevents cleavage by the restriction endonuclease, thus protecting the host DNA from being digested (Kuhnlein and Arber, 1972). RM systems also play roles in recombination, where they mediate integration of horizontally transferred DNA into the host genome (Alm et al., 1999; Nobusato et al., 2000) and have been implicated in genetic isolation and subsequent speciation (Jeltsch, 2003).

RM systems are categorized into four types based on required cofactors and mechanism of activity. Type I RM systems function as pentamer complexes consisting of two restriction endonuclease (R) subunits, two methyltransferase (M) subunits, and a sequence specificity (S) subunit. The target DNA sequences are detected by the two tandem target recognition domains (TRDs) of the S subunit, and each TRD recognizes one half of the two-part target sites (Loenen et al., 2014a). When the complex encounters an unmethylated recognition sequence, the complex binds the DNA and cleaves it at an unpredictable distance from the binding site in an ATP-dependent manner (Murray, 2000). Type II RM systems, by contrast, have restriction endonucleases and methyltransferases that act independently. Type II restriction endonucleases recognize unmethylated sequences (Pingoud et al., 2014). The same recognition sequences are also targeted for modification by the equivalent type II methyltransferases (Murphy et al., 2013). There are also different subgroups of Type II systems, which function differently but primarily as independent RM proteins (Roberts et al., 2003). Type IIG RM proteins, for example, are fusion proteins containing three domains: an N-terminal restriction endonuclease, a central methyltransferase, and C-terminal site specificity (Roberts et al., 2003, 2014; Morgan et al., 2009; Shen et al., 2011). Type III RM systems are two-subunit enzymes consisting of a methyltransferase (*mod*) subunit and a restriction endonuclease (*res*) subunit (Bickle and Kruger, 1993; Rao et al., 2014). The recognition sequences of a Type III RM system are asymmetrical and only modified on one strand of DNA, with cleavage requiring two inversely-oriented, unmethylated recognition sequences (Meisel et al., 1992). Type IV systems consist of genes that target methylated recognition sequences (Roberts et al., 2003; Loenen and Raleigh, 2014).

Although RM systems and DNA methylation have been extensively studied in bacteria, few studies have examined these systems in archaea. Most research on archaeal RM systems has focused on the activity of restriction endonucleases and methyltransferases in hyperthermophilic organisms, such as those belonging to the genus *Pyrococcus* (Chinen et al., 2000; Ishikawa et al., 2005; Watanabe et al., 2006). Studies have also examined cytosine methylation in *Sulfolobus acidocaldarius* (Grogan, 2003), the structure of a type I S subunit in *Methanococcus jannaschii* (Kim et al., 2005), and the activity of a type II methyltransferase in a virus infecting *Natrialba magadii* (Baranyi et al., 2000). However, research on RM systems in other archaeal organisms, and the overall role of these systems, has been limited.

An organism that could prove useful as a model for archaeal RM systems and DNA methylation is *Haloferax volcanii* DS2 (Kuo et al., 1997; Allers and Ngo, 2003; Allers and Mevarech, 2005; Leigh et al., 2011), an archaeal species of the class *Halobacteria* first isolated from the Dead Sea (Mullakhanbhai and Larsen, 1975). *H. volcanii* is useful because it is easy to grow in the lab and has an advanced genetic system (Cline et al., 1989; Allers et al., 2004, 2010; Blaby et al., 2010). Also, the genome of wild-type strain DS2 has been fully-sequenced (Hartman et al., 2010).

Previous research indicated that *H. volcanii* DS2 uses DNA methylation to identify its own DNA from foreign sources. A study on DNA extracted from *H. volcanii* demonstrated that it is resistant to digestion from restriction endonucleases, such as *Xba*I, which recognize motifs containing CTAG (Charlebois et al., 1987). These results indicated that *H. volcanii* DNA was methylated at CTAG tetranucleotide regions; it has since been hypothesized that a putative Type II CTAG methyltransferase HVO_0794 is responsible for this methylation (Hartman et al., 2010). Another study demonstrated that transformation efficiency in *H. volcanii* is greater when using unmethylated DNA from a *dam*^−^ *E. coli* strain (Holmes et al., 1991). The difference in transformation was hypothesized to be the result of cleavage from the putative type IV Mrr restriction endonuclease HVO_0682 (Hartman et al., 2010). This hypothesis was confirmed in another study in which the *HVO_0682* gene was deleted, resulting in higher transformation efficiency when adding methylated DNA (Allers et al., 2010). Overall, this evidence supports the hypothesis that archaeal organisms such as the archaeon *H. volcanii* use RM systems to identify and defend against foreign DNA.

Another potential role for DNA methylation in archaea was uncovered in a recent study on extracellular DNA (eDNA) metabolism in *H. volcanii* (Chimileski et al., 2014). *H. volcanii* was provided with eDNA from different species as a growth substrate and was able to grow using its own DNA as a phosphorus source, but not with herring sperm DNA or methylated *E. coli* DNA. However, *H. volcanii* was able to grow on unmethylated *E. coli* DNA isolated from a *dam*^−^/*dcm*^−^ mutant strain (i.e., without methyltransferase genes). Therefore, methylation may also be used as a means for cells to recognize self and non-self when exploiting eDNA for nutritional purposes and possibly for natural transformation. Determining the methylation patterns of haloarchaeal DNA may shed light on the phenomenon of discriminatory eDNA metabolism.

Recently, a new DNA sequencing technique has been developed which can detect methylated bases, known as single molecule, real-time (SMRT) sequencing. This process determines the sequence of DNA as a new strand is synthesized in real time, and the kinetic signals of incorporated bases can also be measured (Flusberg et al., 2010). Unique kinetic signals produced at modified bases during strand synthesis are used to detect methylation patterns of sequenced DNA. This process has been used to sequence the genomic methylation patterns, or methylomes, of several different bacterial species (Fang et al., 2012; Murray et al., 2012; Lluch-Senar et al., 2013; Furuta et al., 2014; Krebes et al., 2014). In this study, we used SMRT sequencing to characterize the methylomes of H. *volcanii* H26, a laboratory strain derived from wild-type strain DS2, and a derivative strain in which the gene *HVO_A0006* was deleted. HVO_A0006 is annotated in the *H. volcanii* genome as an adenine methyltransferase and is located on the native replicon pHV4 (Hartman et al., 2010). It is predicted to encode a protein that is 219 amino acids in length and with a molecular weight of 24.794 kDa. HVO_A0006 was selected for further characterization because, despite being annotated as a methyltransferase, it is not recognized as an RM protein by the RM database REBASE (Roberts et al., 2014).

## Materials and methods

### Strains and growth conditions

Descriptions of all strains and plasmids used in this study are provided in Table 1. *E. coli* strains were grown in Lysogeny Broth (LB; Ampicillin was added at 100 μg mL^−1^ when necessary). *H. volcanii* strains were grown in Hv-YPC (rich medium) or Hv-CA (selective rich medium) developed by Allers et al. (2004) per instructions in the *Halohandbook* (Dyall-Smith, 2008). Uracil (50 μg mL^−1^), tryptophan (50 μg mL^−1^), and 5-fluoroorotic acid (at 50 μg mL^−1^) were added to the media as needed for Δ*pyrE2* and Δ*trpA* strains. All *H. volcanii* cultures were incubated at 42°C and shaken at 200 rpm unless otherwise specified.

**Table 1:** **Strains and plasmids used in this study**.

**Strain/plasmid name**	**Description**	**Source**
**STRAINS**		
*Escherichia coli* HST08	*E. coli* cloning strain	Clontech, Cat. # 636763
*Escherichia coli dam-/dcm-*	Used for producing unmethylated plasmids for *Haloferax* transformations	Clontech Cat # C2925H
*Haloferax volcanii* DS2	Wild-type	Mullakhanbhai and Larsen, 1975
*Haloferax volcanii* H26	Δ*pyrE*2; laboratory strain derived from DS2	Bitan-Banin et al., 2003
*Haloferax volcanii* H53	Δ*pyrE*2/Δ*trpA*; derived from H26	Allers et al., 2004
*Haloferax volcanii* H26 Δ*HVO_A0006*	*HVO_A0006* deletion strain; derived from H53	This study
**PLASMIDS**		
pTA131	Vector used to create pΔ*HVO_A0006* gene deletion. Contains ampicillin resistance for selectivity, *lacZ* cloning site for blue-white screening, and *pyrE2* marker for *H. volcanii* screening	Allers et al., 2004
pΔ*HVO_A0006*	pTA131 vector construct with flanking region insert used to knockout *HVO_A0006*	This study

### Deletion of *HVO_A0006* gene

**Figure 1:**
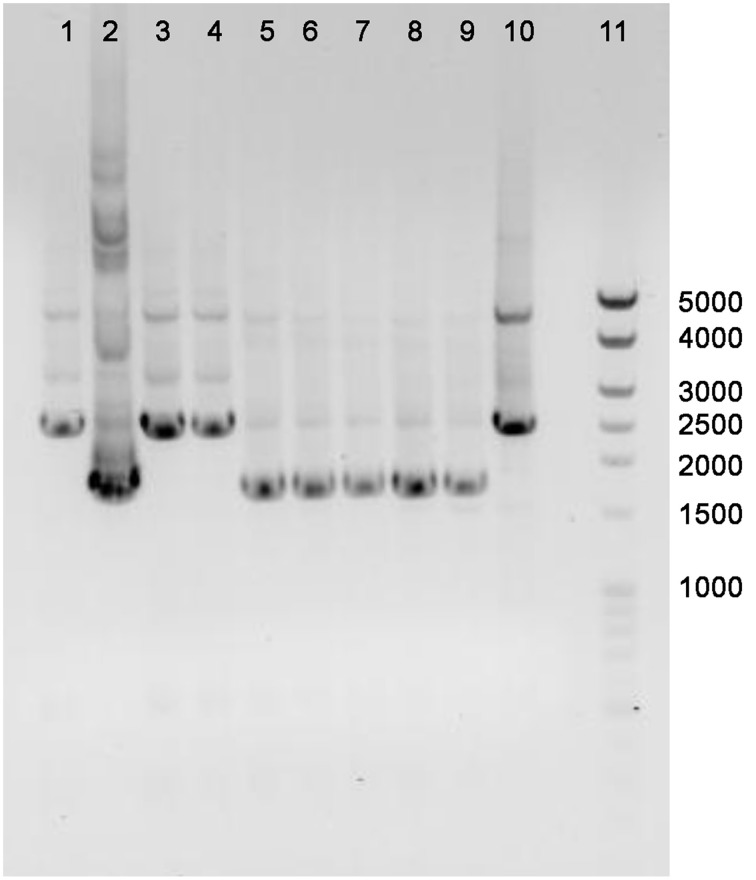
**PCR confirmation of *HVO_A0006* deletion strain**. The template DNA amplified was from *H. volcanii* DS2 (Lane 1), pΔ*HVO_A0006* (lane 2; as a positive control), unsuccessful pop-outs (lanes 3, 4, and 10) and successful pop-out colonies (lanes 5–9). A Mid-Range DNA ladder (Fisher Scientific) is shown in Lane 11.

The annotated adenine methyltransferase gene *HVO_A0006* (accession number ADE01899) was selected for deletion in *H. volcanii* strain H53. The gene deletion strategy used in this study was modified from the methodology previously developed (Blaby et al., 2010) and uses the In-Fusion HD Cloning Kit (Clontech). The primer sequences used in this study are listed in Table 2. *H. volcanii* strain H53 was then transformed with pΔ*HVO_A0006* using the PEG-mediated transformation of haloarchaea protocol from Cline et al. (1989), Bitan-Banin et al. (2003), Allers et al. (2004), and Blaby et al. (2010). The transformed cells were then plated on Hv-CA agar media without uracil and incubated for 5–7 days at 42°C. Colony PCR was performed using forward and reverse M13 primers to screen for pop-ins. The positive colonies from the pop-in screen were plated on Hv-CA plates with 50 μg mL^−1^ 5-FOA and 50 μg mL^−1^ uracil to obtain pop-outs. Final pop-outs were confirmed through PCR (see primers in Table 2) as visualized through gel electrophoresis (Figure 1).

**Table 2:** **Oligonucleotide primers used in this study**.

**Primer name**	**Primer sequence**	**Primer description**
HVO_A0006 FRIF	5′-CGG GCC CCC CCT CGA GTC AAG CAG TAC CTC AAC ACG GAA CA-3′	Used to amplify the flanking regions of *HVO_A0006*, with *Xho*I and *Xba*I
HVO_A0006 FR1R	5′-ATT CGA TAT CAA GCT GTC CTC AAG GAC GGC CTG CA-3′	
HVO_A0006 FR2F	5′-GAC GCG TTG ATA TCC CGA AGA ATC CAG TTG CTG TCT GTT G-3′	
HVO_A0006 FR2R	5′-GGA TAT CAA CGC GTC GGC ATT ATG CAA TTC-3′	
M13F	5′-GTA AAA CGA CGG CCA GT-3′	Primers used to amplify the flanking regions of *HVO_A0006* inserted into the multiple cloning site of pTA131. Used for the screening of recombinant plasmids
M13R	5′-AGG AAA CAG CTA TGA CCA T-3′	

### DNA preparation for PacBio sequencing

To extract DNA from *H. volcanii* strains H26 and Δ*HVO_A0006* for PacBio SMRT sequencing, 5 mL of cell cultures in log phase were pelleted and resuspended in 5 mL DNA buffer (10 mM Tris-HCl, pH 8.0) to lyse the cells. Lysates were treated with 100 μg mL^−1^ RNase A and incubated overnight at 42°C to degrade RNA. Proteinase K 10 mg mL^−1^ was added to the lysates to a final concentration of 50 μg mL^−1^ and incubated at 37°C for 1 h to degrade protein, followed by ethanol precipitation. Three rounds of phenol/chloroform extraction were then performed to purify the DNA (until no interphase was visible). The top aqueous layer from each tube was removed and a final ethanol precipitation was performed as described above. The 260/280 ratio and 260/230 ratio for each DNA sample were measured for purity (H26: 260/280 = 1.83, 260/230 = 2.28; Δ*HVO_A0006*: 260/280 = 1.78, 260/230 = 2.27).

### PacBio SMRT sequencing

The methylation patterns of DNA extracted from *H. volcanii* H26 and the Δ*HVO_A0006* strain were sequenced using PacBio SMRT sequencing. The prepared samples were processed by the Keck Sequencing facility of the Yale School of Medicine for analysis using PacBio SMRT sequencing. The SMRT method is described in detail in the PacBio manual “Detecting DNA Base Modifications: SMRT Analysis of Microbial Methylomes” (http://www.pacb.com/pdf/TN_Detecting_DNA_Base_Modifications.pdf). Using an estimated input DNA size range of ~4000 kb, 500–800 bp libraries were prepared for each strain and were run in one SMRT cell, yielding ~60x coverage for H26 and ~80x coverage for Δ*HVO_A0006*. Analysis of the methylated bases and motifs in each strain was performed using the “RS_Modification_and_Motif_Analysis.1” program in SMRT Portal under default parameter settings, with the *H. volcanii* DS2 genome used as a reference (Hartman et al., 2010). The “motifs.gff” output files for both strains are available in Supplementary Data (Supplementary Data Sheet 1 for H26 and Supplementary Data Sheet 2 for Δ*HVO_A0006*).

### Protein domain architecture analysis and multiple alignment

Homologs of the HVO_A0006 protein were identified through a blastp search (Altschul et al., 1990) of the non-redundant protein database (based on an *E*-value threshold for inferring homology of 1e-4). Structural Classification of Proteins (SCOP) database superfamilies (Gough and Chothia, 2002) and sequence features shown in domain architecture diagrams were detected with InterProScan (Quevillon et al., 2005). Multiple alignments were generated with Clustal Omega (Sievers et al., 2011). Secondary structure predictions for the multiple alignment were made using PRALINE (Heringa, 1999) and PSIPRED (Jones, 1999). PD-(D/E)XK motifs were identified with the PD-EXK web server (Laganeckas et al., 2011).

## Results

### Characterization of DNA methylation in *Haloferax volcanii* H26

In order to characterize the methylome of *H. volcanii*, the genome of laboratory strain H26 (Table 1) was sequenced via SMRT sequencing. The data from the SMRT sequencing analysis for H26 is summarized in Table 3. SMRT sequencing of *H. volcanii* H26 identified two modification motifs for H26: one in which cytosine was methylated (m^4^C) and another in which adenine was methylated (m^6^A). The m^4^C motif was identified as C^m4^TAG and the m^6^A sequence motif was identified as GCA^m6^ BN_6_VTGC. These two motifs are also the same as those predicted for *H. volcanii* DS2, the parental strain of H26, in REBASE (Roberts et al., 2014). The CTAG motif was identified 1342 times in the H26 genome, and methylation was detected at 374 of these motifs (28% methylation). The GCABN_6_VTGC motif occurred 410 times in the H26 genome, and 316 of the copies were detected to have methylated bases (77%).

**Table 3:** **DNA methylation patterns detected for *H. volcanii* H26 and Δ*HVO_A0006***.

**Strain**	**H26**		**Δ*HVO_A0006***	
**Motif**	**GCA^m6^ BN_6_VTGC**	**C^m4^TAG**	**GCA^m6^ BGN_5_VTGC**	**C^m4^TAG**
Methylated position	3	1	3	1
Methylation type	m^6^A	m^4^C	m^6^A	m^4^C
Number of methylated motifs	316	374	141	662
Number of motifs in genome	410	1342	160	1342
Percent of methylated motifs	77	28	88	49
Mean modification QV^*^	57.0	49.7	70.9	60.0
Mean motif coverage	30.7	48.4	42.9	62.7

* *Modification QV refers to level of confidence that a base is methylated. A QV of 30 or higher is considered significant*.

### Deletion of *HVO_A0006* increases specificity of m^6^A motif and reduces m^6^A methylation

In order to determine the effect of HVO_A0006 on genome wide methylation, the chromosome of strain H53 with the *HVO_A0006* gene deleted (Δ*HVO_A0006*) was sequenced via SMRT sequencing. The data from the SMRT sequencing analysis is summarized in Table 3 for Δ*HVO_A0006*. In Δ*HVO_A0006*, the C^m4^TAG motif was identified like in H26, although the sequencing coverage was much higher and more methylated motifs were detected. However, the m^6^A motif differed significantly for the knockout strain. First, the methylated m^6^A motif in the Δ*HVO_A0006* strain was more specific than in its H26 counterpart, with one of the unspecified nucleotides in the H26 sequence (N) being identified as a guanine (G) in the Δ*HVO_A0006* strain. The resulting methylation motif from the Δ*HVO_A0006* strain is GCA^m6^ BGN_5_VTGC. Thus, the m^6^A sequence became more specific with the deletion of *HVO_A0006*. Secondly, the total number of detected motifs, and the number of detected methylated m^6^A motifs in comparison to the reference genome also decreased when the *HVO_A0006* gene was deleted. In Δ*HVO_A0006*, the total number of detected GCABGN_5_VTGC motifs was 160, 61% less than H26, and the number of methylated GCA^m6^ BGN_5_VTGC sequences was 141, a decrease of 55% from H26. This decrease was due to GCABHN_5_VTGC sequences not being included in the motif total for Δ*HVO_A0006*, since those sites were not methylated in that strain. This should also explain why the percentage of methylated m^6^A motifs is higher in Δ*HVO_A0006* (88%) compared to H26 (77%) even though Δ*HVO_A0006* has fewer methylated m^6^A motifs. Differences were also observed in the number of methylated m^4^C sites detected, with Δ*HVO_A0006* exhibiting an increase in the number of methylated C^m4^TAG sequences compared to H26. However, this change was likely the result of a sequence coverage artifact, since the motif coverage for CTAG was higher in Δ*HVO_A0006* (62.7) than in H26 (48.4). It is also important to note that SMRT sequencing only reports coverage of a motif above a certain modification quality value (QV) score. For both the adenine and cytosine motifs, the knockout strain QV was higher overall with an average value of 60.9 as compared to 49.7 for the H26 strain. It is highly plausible that the cytosine motifs were equally methylated but some of the methylated bases did not reach the QV threshold and were therefore not counted. Therefore, the discrepancy in methylated m^4^C sites is unlikely to be caused by the deletion of the *HVO_A0006* gene. Overall, the results are conclusive that deletion of *HVO_A0006* reduces m^6^A methylation in *H. volcanii*.

### HVO_A0006 is a fragment homologous to larger, multi-domain RM proteins

**Figure 2:**
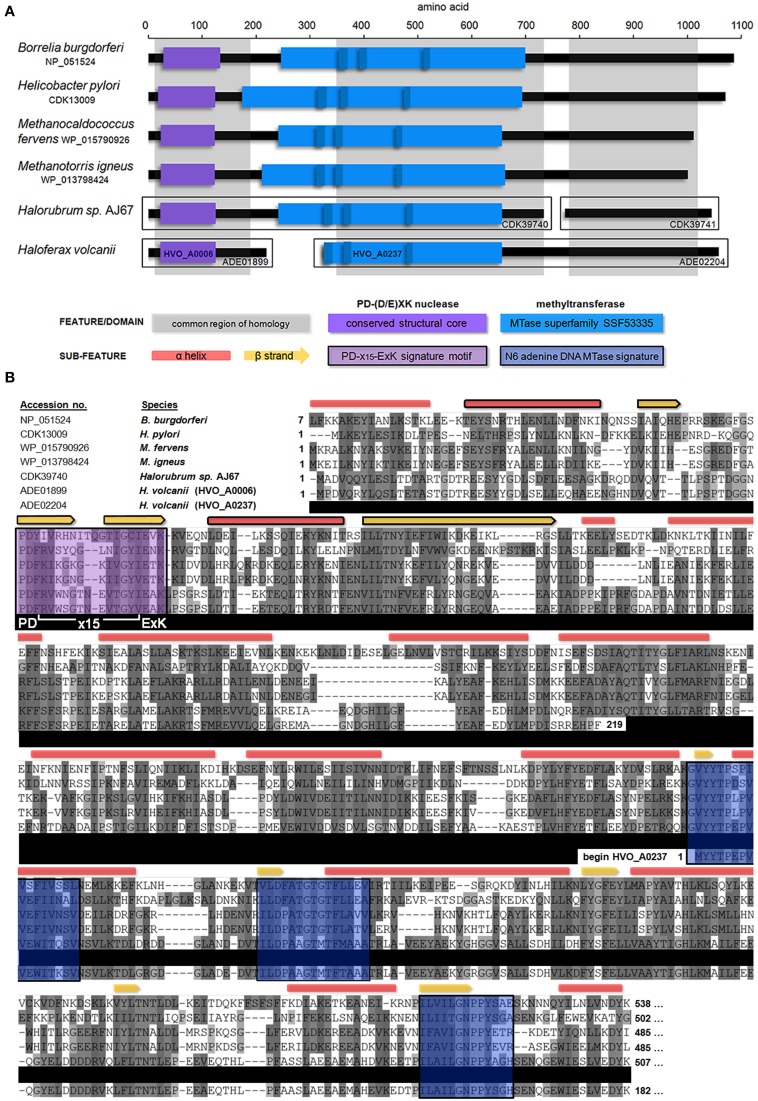
**Domain architecture and multiple alignment of Type IIG homologs**. **(A)** The position of predicted domains present within HVO_A0006 (accession number ADE01899) homologs is shown, including experimentally characterized DNA methyltransferases from *B. burgdorferi* and *H. pylori* and several annotated methyltransferases from archaeal species. The common regions of homology between all proteins are shown in gray. Detected s-adenosyl-L-methionine-dependent methyltransferase superfamily domains (SSF53335) are shown in light blue, along with N6 adenine specific DNA methlytransferase signatures (IPR002296; dark blue). The conserved structural core (αβββαβ) of a PD-(D/E)XK nuclease domain is shown in purple. All sequences shown contained a PD-(D/E)XK motif with a confidence score of 1.0 (Laganeckas et al., 2011). **(B)** Multiple alignment of HVO_A0006 homologs. Predicted DNA methlytransferase signatures (blue) and PD-(D/E)XK signatures (purple) from part A are highlighted. Conserved secondary structure predictions are shown above: as red boxes for α-helices and yellow arrows for β-sheets. Components of the conserved αβββαβ core of the predicted PD-(D/E)XK nuclease domain shown in part A are outlined in black. Amino acid shading represents Clustal sequence similarity. All sequences other than HVO_A0006 are truncated (see Figure S1 for entire alignment).

A blastp search indicated that HVO_A0006 is a rare protein among the sequenced *Halobacteria*. The only significant hit within this group was an annotated adenine specific DNA methyltransferase domain protein belonging to *Halorubrum sp*. AJ67 (Table 4). Out of the top 10 significant hits only a few belonged to archaeal species and the rest were bacterial (Table 4). The blastp analysis also found homologs experimentally characterized as methyltransferases from *Borrelia burgdorferi* and *Helicobacter pylori* (Rego et al., 2011; Krebes et al., 2014). However, those homologs are much larger than HVO_A0006, spanning between 700 and 1000 amino acids (Figure 2). A reciprocal blastp of those hits against the *H. volcanii* DS2 and *Halorubrum sp*. AJ67 genomes revealed there are additional homologs. One is *H. volcanii* gene *HVO_A0237* (accession number ADE02204), also annotated as an adenine methyltransferase, listed in REBASE as HvoDSORF237P, and located on pHV4 231 genes downstream from *HVO_A0006*. Another identified homolog is a small annotated adenine methyltransferase in *Halorubrum sp*. AJ67.

**Table 4:** **Top 10 blastp hits for *H. volcanii* HVO_A0006**.

**Species name**	**Accession number**	**Annotated function**	***E*-value**	**Predicted amino acid length**	**Reciprocal blastp *E*-value**
*Halorubrum* sp. AJ67	CDK39740	adenine specific DNA methyltransferase domain protein	7e-120	734	2e-123
*Peptococcaceae* bacterium SCADC1_2_3	KFD42170	DNA methyltransferase	3e-59	1040	1e-62
Unclassified *Atribacteria*	WP_018206408	hypothetical protein	3e-56	1041	9e-60
*Aerophobetes* bacterium SCGC AAA255-F10	WP_029964223	DNA methyltransferase, partial	2e-55	1024	7e-59
*Smithella* sp. SCADC	KFO67113	DNA methyltransferase	1e-54	1049	4e-58
Unclassified *Aminicenantes*	WP_020261071	hypothetical protein	3e-54	1034	1e-57
*Aminicenantes* bacterium SCGC AAA252-G21	WP_020260848	hypothetical protein, partial	3e-54	980	6e-59
*Atribacteria* bacterium SCGC AAA255-N14	WP_029955983	DNA methyltransferase	4e-54	1037	1e-57
*Melioribacter roseus* P3M-2	WP_014855429	adenine specific DNA methyltransferase	7e-54	1042	2e-57
*Cloacimonetes* bacterium JGI OTU-1	WP_024562979	DNA methyltransferase	1e-52	1041	5e-56

In REBASE, homologs of HVO_A0006 are predicted to belong to the Type IIG subgroup. A multiple sequence alignment of HVO_A0006 (Figure 2) and several of its homologs indicate that the shared sequence identity occurs only in the N-terminal, putative endonuclease region. The homologs of HVO_A0006 contain known methyltransferase features in their central regions, including the s-adenosyl-L-methionine-dependent methyltransferse superfamily domain (SSF53335) within the SCOP database and predicted N6 adenine specific DNA methyltransferase signatures (IPR002296; Figure 2A), but these do not overlap with HVO_A0006 in the alignment. The N-terminal region of HVO_A0237 contains this methyltransferase domain, but it aligns with the central region of the other sequences, not with HVO_A0006. The smaller annotated adenine methyltransferase in *Halorubrum sp*. AJ67 also did not align with HVO_A0006, or with the larger annotated methyltransferase in the same *Halorubrum* strain, but instead aligned with the C-terminal region of the other homologs in Figure 2 (see also Figure S1). Overall, these results indicate that HVO_A0006 is not an adenine methyltransferase, as it was annotated, but is instead a fragment homologous to the N terminus of Type IIG RM fusion proteins.

### HVO_A0006 contains a conserved PD-(D/E)XK nuclease motif

Analysis of the HVO_A0006 sequence for endonuclease signatures revealed that it contains a PD-(D/E)XK nuclease motif (PD^60^-X_14_-E^75^AK^77^), a Mg^2+^-dependent catalytic motif common to many restriction endonucleases (Kosinski et al., 2005). The active sites of this motif (D^60^, E^75^, and K^77^) are conserved in all homologs in the multiple sequence alignment which align with HVO_A0006 (Figure 2B). Analysis of these sequences in the alignment using the PD-EXK web server (Laganeckas et al., 2011) predicted each sequence belonged to a PD-(D/E)XK family with a probability of 1.0. Secondary structural analysis also demonstrated that the region containing the PD-(D/E)XK motif in the aligned sequences all share an αβββαβ core structure common to this domain family (Venclovas et al., 1994; Kinch et al., 2005). Overall, this evidence indicates that HVO_A0006 is a PD-(D/E)XK nuclease family protein.

### *HVO_A0006* and *HVO_A0237* are flanked by predicted integrase and transposase genes

**Figure 3:**
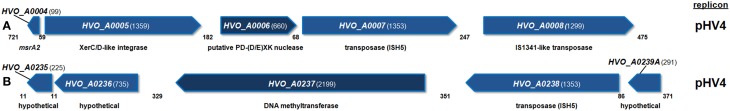
**Diagram of the genomic neighborhood for *H. volcanii HVO_A0006* (A) and *HVO_A0237* (B)**. Genes are depicted along with two upstream and downstream flanking genes, with annotated functional predictions below. Gene sizes and intergenic spaces are shown in nucleotides. Both regions shown are found on replicon pHV4.

The gene neighborhoods of *HVO_A0006* and *HVO_A0237* were examined. Analysis of the surrounding genomic region of *HVO_A0006* (Figure 3A) revealed that the gene is flanked by three transposition genes: a XerC/D-like integrase and two putative transposases. A putative transposase was also found directly upstream of *HVO_A0237* (Figure 3B). This predicted protein (HVO_A0238) is the same length and 100% identical to the ISH5 transposase adjacent to HVO_A0006: HVO_A0007 (Figure 3).

## Discussion

Previous research on DNA methylation has focused primarily on eukaryotic and bacterial organisms. The few studies which have examined DNA methylation and RM systems in archaeal organisms have concentrated on a few restriction endonucleases and methyltransferases, with no emphasis on overall genomic methylation (Baranyi et al., 2000; Chinen et al., 2000; Grogan, 2003; Ishikawa et al., 2005; Kim et al., 2005; Watanabe et al., 2006). In this study, we characterized genome-wide DNA methylation patterns of an archaeal organism. We used *H. volcanii* due to its developed genetic system and ease of growth, which allowed us to examine the effect of deleting one of the seven annotated methyltransferases in the *H. volcanii* genome.

The SMRT sequencing analysis detected two motifs, which are modified throughout the *H. volcanii* H26 genome: a C^m4^TAG motif and a GCA^m6^BN_6_VTGC motif. The presence of methylated CTAG motifs is consistent with observations from previous restriction digest experiments, and the putative Type II CTAG methyltransferase HVO_0794 may be responsible for modifying these motifs (Charlebois et al., 1987). The GCA^m6^BN_6_VTGC motif resembles the type of sequence targeted by Type I RM systems, which typically consist of two partial sequences separated by a gap of unspecified nucleotides (Loenen et al., 2014a). The only Type I RM system predicted in *H. volcanii*, according to the RM database REBASE, is the operon *HVO_2269-2271* named *rmeRMS* (Roberts et al., 2014). Therefore, we predict that the Type I complex encoded by the *rmeRMS* operon is responsible for at least some of the modifications detected by the SMRT sequencing. Further studies would need to be performed to confirm the role of these putative RM systems in methylating the identified motifs, as well as determining the role of the other annotated methyltransferase genes in the *H. volcanii* genome.

The gene *HVO_A0006* was selected for investigation via gene knockout in this study because in genomic analysis it was annotated as an adenosine specific methyltransferase (Hartman et al., 2010). The results indicate that the *HVO_A0006* gene has an effect on m^6^A methylation in *H. volcanii* according to the motif analysis produced by SMRT sequencing. This is evidenced by the observed change in adenine motif recognition between the H26 strain, which was GCA^m6^ BN_6_VTGC, and the *HVO_A0006* knockout, which was GCA^m6^BGN_5_VTGC. Our conclusion is also supported by the reduction in the total amount of motifs recognized, with the knockout strain exhibiting a 61% decrease in the total number of recognized motifs compared to the parental strain and a 55% reduction in the number of methylated motifs. These data appear to support the genome annotation of *HVO_A0006* as an adenine methyltransferase. However, the amino acid alignment and domain architecture analysis of the protein and its homologs reveals that HVO_A0006 is only homologous to the N-terminal region of characterized and predicted Type IIG RM fusion proteins, which typically contain a restriction endonuclease domain. Analysis of the conserved region in HVO_A0006 and its homologs revealed the presence of a PD-(D/E)XK nuclease motif with conserved active sites and secondary structure. While methyltransferase domains were detected in homologs of HVO_A0006, they do not correspond to the region of shared sequence identity with HVO_A0006. Therefore, HVO_A0006 likely functions as a nuclease, not a complete methyltransferase as its annotation suggests.

We hypothesize that HVO_A0006 influences adenine methylation patterns through interaction with another RM protein or system in *H. volcanii*. One possible candidate for interaction is the Type I RM system encoded by *rmeRMS*. In Type I RM systems, the M subunits are known to interact with each other to coordinate restriction and modification activity in the Type I complex. This interaction primarily occurs via N-terminal helical regions in the M subunits, which coordinate to detect adenine methylation when the complex binds to the target sequence and mediate methylation if the site is hemimethylated (maintenance methylation), or cleavage if the site is unmethylated (Kelleher et al., 1991). Mutation of this N-terminal region alters the activity of the Type I complex to *de novo* methylation (modification of unmethylated target sites) rather than maintenance methylation, indicating that changes to molecular communication in the Type I complex can alter RM activity (Kelleher et al., 1991). In HVO_A0006, we detect a helical region in the C-terminal region of the protein. This region is likely part of the helical connector domain that links the restriction endonuclease and methyltransferase domains in Type IIG RM proteins (Shen et al., 2011). The Type IIG RM systems are hypothesized to be evolutionarily related to the Type I systems, and this helical connector domain is predicted to be homologous to the N-terminal helical region of the Type I M subunits, with a similar function of molecular communication between the methyltransferase and restriction endonuclease domains (Nakonieczna et al., 2009). Therefore, it is possible that HVO_A0006 uses its partial helical domain to interact with the Type I RmeRMS complex and acts as an R subunit, thus altering the restriction and modification activity of the complex and resulting in the different methylation patterns seen in the parental strain of *H. volcanii* compared to the *HVO_A0006* knockout. However, since the RmeRMS system already has an R subunit encoded in the operon, any possible interaction between HVO_A0006 and the Type I complex would be complicated by competition with the native subunit.

Another possible candidate protein for interaction with HVO_A0006 is HVO_A0237 which are both located on the native plasmid pHV4. The multiple alignment analysis (Figure 2) indicates that HVO_A0237 is also a homolog of a Type IIG system, but it is missing an N-terminal restriction endonuclease domain. This absent region might be supplied by HVO_A0006, which interacts with HVO_A0237 via the helical connector region to form a complete Type IIG RM enzyme, and forms a split protein complex, rather than a fused one as is typical of these systems. Previous analysis has demonstrated that the activity of a Type IIG protein is coordinated by the interaction between the restriction endonuclease domain and the other two regions of the enzyme (Shen et al., 2011). Therefore, deletion of the *HVO_A0006* gene might compromise the integrity of the required Type IIG protein-protein interactions and thus prevent methylation by the remaining methyltransferase encoded by *HVO_A0237*, resulting in the change in adenine methylation observed in the deletion mutant. Complicating this proposed mechanism is that motifs modified by Type IIG systems do not typically resemble Type I motifs. For example, the motif modified by the *H. pylori* homolog is GGWTAA^m6^ (Krebes et al., 2014). However, some Type IIG proteins in *Campylobacter jejuni* have been reported to use S subunits similar to those found in Type I S RM systems (Furuta et al., 2010). Further studies will be needed to characterize the interactions between HVO_A0006 and other RM systems.

Based on a multiple alignment and the presence of integrase and transposition genes upstream and downstream of the gene, we propose that *HVO_A0006* is likely the result of a gene transfer event of a Type IIG RM in which a gene fragment (the N-terminal restriction endonuclease region) of the whole gene was acquired. The other regions of the Type IIG RM gene were likely also acquired or were the result of internal genomic rearrangements from a once intact gene in *H. volcanii*, since *HVO_A0237* is missing an N-terminal restriction endonuclease region, but is homologous to the same Type IIG RM genes as *HVO_A0006*. A similar event also appears to have occurred with a Type IIG RM gene in *Halorubrum* sp. AJ67, with the C-terminal site specificity region fragmenting from the restriction endonuclease and methyltransferase regions. This is consistent with previous findings of gene transfer that have resulted in gene fragmentation (Kobayashi et al., 1999; Chan et al., 2009). These RM systems are highly mobile, and gene transfer events of RM domains and subunits likely results in the formation of new fusion proteins, when the domains are transferred next to each other, as well as new independent RM proteins via fragmentation.

Using SMRT sequencing, this study was able to characterize the methylome of the archaeal organism *H. volcanii*. We were also able to demonstrate that an annotated methyltransferase gene, *HVO_A0006*, affects adenine methylation patterns in the *H. volcanii* genome, although it is likely a PD-(D/E)XK nuclease family protein that interacts with another RM protein or system. Future SMRT sequencing studies utilizing a methyltransferase null mutant could help provide more precise characterization of methyltransferase genes and provide a more detailed picture of DNA methylation in *H. volcanii* and the role of RM systems in the organism.

### Conflict of interest statement

The Review Editor Antonio Ventosa declares that, despite having published with author Thane Papke, the review process was handled objectively and no conflict of interest exists. The authors declare that the research was conducted in the absence of any commercial or financial relationships that could be construed as a potential conflict of interest.
