# Supplementary material for: Genome-wide DNA methylation analysis of Haloferax volcanii H26 and identification of DNA methyltransferase related PD-(D/E)XK nuclease family protein HVO_A0006
Source: Front Microbiol. 2015 Apr 8;6:251. doi: 10.3389/fmicb.2015.00251 (PMC4389544; doi:10.3389/fmicb.2015.00251)
Supplement: Supplementary file 3 [file Image1.PDF]

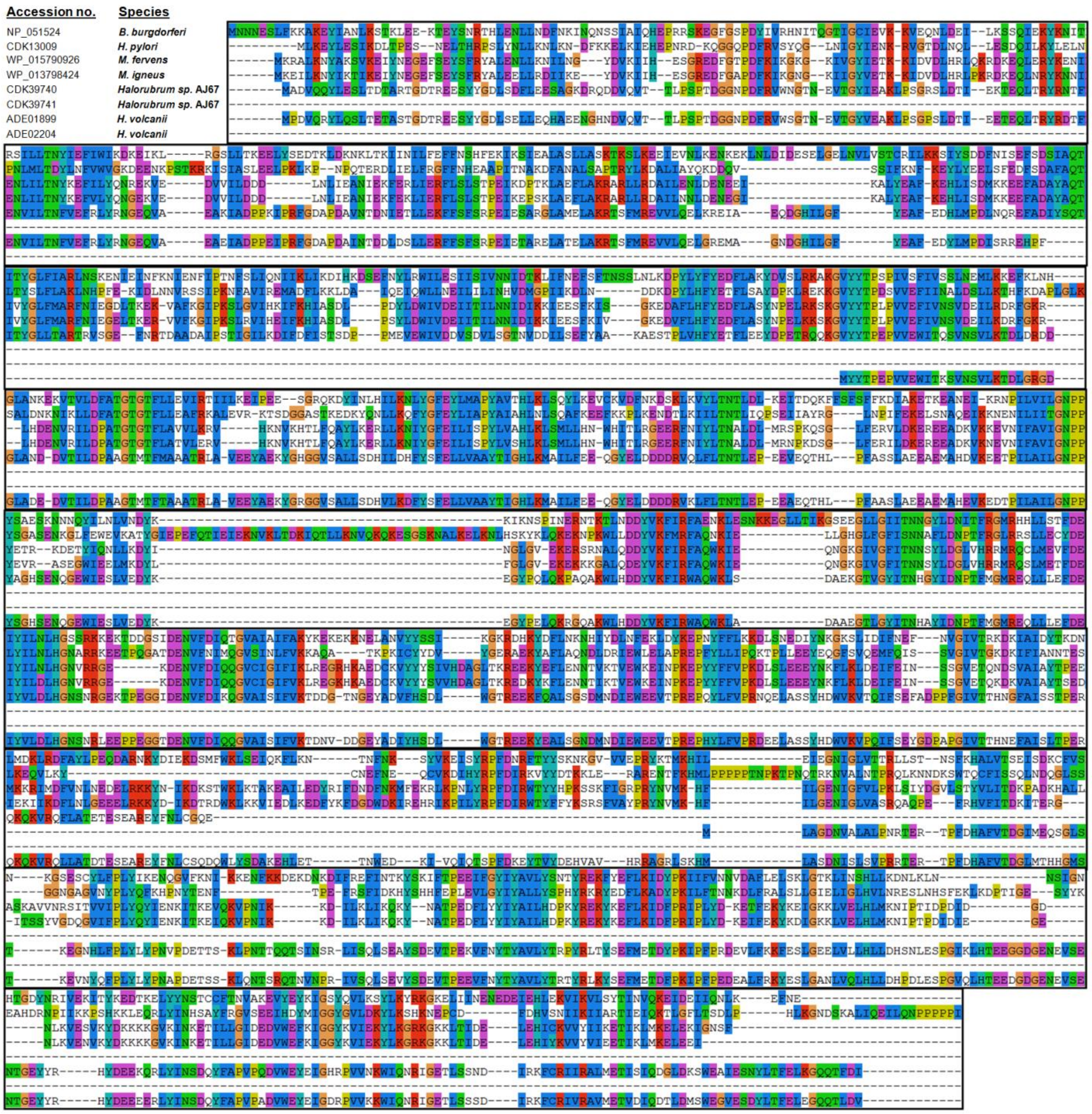

Figure S1. Full length multiple alignment of HVO_A0006 homologs. Amino acid shading represents Clustal sequence similarity.
